# Beneficial Effects of Pulse Methylprednisolone Therapy in COVID-19 Patients at Risk of Hyperinflammatory Response

**DOI:** 10.7759/cureus.90549

**Published:** 2025-08-20

**Authors:** Jeevitha Dandiganahalli Channappa, Jagadeesh Kumar

**Affiliations:** 1 Department of Surgery, Apollo Hospitals, Bengaluru, IND; 2 Department of Pulmonology, Apollo Hospitals, Bengaluru, IND

**Keywords:** acute respiratory distress syndrome, covid-19, hospitalization, hyperinflammatory response, oxygen, pandemic, pulse methylprednisolone therapy

## Abstract

Rationale: Pulse methylprednisolone therapy can prevent cytokine storm in patients with severe COVID-19. The beneficial effects of pulse methylprednisolone therapy in terms of survival and duration of hospitalization in severe COVID-19 patients have not been explicitly demonstrated. Although prior studies have reported favorable outcomes, many were limited by small sample sizes, inconsistent steroid regimens, or lack of control groups. This study explicitly evaluates the efficacy of pulse methylprednisolone through matched-group comparison with well-defined biochemical and clinical endpoints.

Objective: This study was performed to evaluate the efficacy of pulse methylprednisolone therapy in severe COVID-19 patients at risk of hyperinflammatory response. Efficacy was measured by early mortality (Day 3), discharge rate, peripheral capillary oxygen saturation improvement, oxygen requirement reduction, and changes in inflammatory markers (C-reactive protein, CRP; interleukin-6, IL-6; D-dimer; ferritin; and LDH).

Methods: This was a retrospective observational study. Ninety patients with confirmed COVID-19, who were at risk of a hyperinflammatory response and had elevated serum inflammatory markers, as well as critically ill COVID-19 patients, were treated with pulse methylprednisolone therapy. This therapy involved administering doses of 125, 250, and >250 mg twice daily, tailored according to severity indicators and IL-6 thresholds, in addition to standard treatment for a duration of three days (Group A). Ninety age-, gender-, and disease severity-matched COVID-19 patients were taken as controls and were treated with standard treatment alone (Group B). The primary outcomes were the mortality rate at Day 3 and the duration of hospitalization.

Results: There were significant differences in the mortality rate at Day 3 (16.7% vs. 52.2%, p < 0.05) and the duration of hospitalization, 10.9 (3-22) vs. 15.3 (1-30) days, between Groups A and B. Pulse methylprednisolone therapy significantly improved the recovery (discharge) rate (83.3% vs. 47.8%), oxygen saturation (92% vs. 86%), and oxygen requirement (10 vs. 12 L/minute) compared with standard treatment alone at the end of treatment and discharge/death. There were significant improvements in inflammatory markers, including CRP, D-dimer, IL-6, ferritin, and lactate dehydrogenase levels, in Group A compared with those in Group B. No immediate adverse events with pulse methylprednisolone therapy were reported; formal monitoring protocols were limited due to retrospective design. Long term adverse effects such as secondary infections or hyperglycemia were not assessed due to study design.

Conclusions: Adjunct pulse methylprednisolone therapy significantly improved mortality rate, duration of hospitalization, enhanced recovery rate, decreased oxygen requirement, and improved laboratory parameters compared to standard treatment alone in patients with severe COVID-19 at risk of hyperinflammatory response. These benefits may be attributed to pulse methylprednisolone’s rapid onset of action and potent nongenomic anti-inflammatory effects, which allow for faster suppression of key cytokines such as IL-6 and CRP. This mechanistic advantage may explain its superior efficacy over conventional corticosteroids like dexamethasone in mitigating the cytokine storm associated with severe COVID-19. Further prospective studies are warranted to confirm these findings and assess long-term safety outcomes.

## Introduction

The world witnessed the COVID-19 pandemic in the past few years (2019-2022) [1]. The clinical spectrum of COVID-19 ranges from fever, dry cough, fatigue, myalgia, and mild respiratory tract symptoms to a severe form, including shortness of breath, pneumonia, and acute respiratory distress syndrome (ARDS) [2]. In the early phase of the disease, an effective immune response eliminates viral replication and prevents disease progression to the hyperinflammation phase. If the immune system does not control the infection, the disease enters the inflammatory phase, where elevated inflammatory markers are produced by innate immune cells, leading to cytokine storm [3]. In this phase, pulmonary fibrosis, dyspnea, drop in peripheral capillary oxygen saturation (SpO_2_), and systemic injuries are noted, resulting in ARDS and death [4].

Various treatment options have been tried for the management of COVID-19 [5]. As ARDS is the main cause of death in COVID-19 [6], it is thought that corticosteroids and immunosuppressive agents can ameliorate the hyperinflammatory phase and prevent cytokine storm and ARDS induction [7]. The Randomized Evaluation of COVID-19 Therapy trial showed that dexamethasone could improve the 28-day mortality rate in patients with COVID-19 who are on oxygen support [8]. A handful of other evidence is also available, supporting the use of corticosteroids in COVID-19; however, there is a lot of heterogeneity in the doses and types of corticosteroids used across various studies [9].

Methylprednisolone is an intermediate-acting, potent, anti-inflammatory agent having a low risk of sodium and water retention. It is administered at a dose of 20-30 mg/kg of body weight (500-1,000 mg/m^2^ of body surface area) per pulse up to a maximum dose of 1,000 mg [10]. Pulse methylprednisolone therapy has a profound anti-inflammatory effect, and this could be beneficial in patients with COVID-19 who are at risk of hyperinflammatory response and cytokine storm [11]. Unlike standard-dose dexamethasone (6 mg/day), pulse methylprednisolone induces rapid nongenomic immunosuppression, promoting apoptosis of hyperactivated immune cells, potentially halting cytokine storm progression more effectively [11]. This treatment could reduce the requirement of long-term corticosteroids, and hence, the resulting adverse consequences [12]. Further, there is a lower risk of a prolonged suppressive effect on the hypothalamic-pituitary axis with this treatment [13]. Hence, we evaluated the efficacy of pulse methylprednisolone therapy in severe COVID-19 patients at risk of hyperinflammatory response through matched-group comparison with well-defined biochemical and clinical endpoints. While studies have shown increased survival with high-dose corticosteroids, our study adds to the literature by specifically analyzing early mortality (Day 3), matched control outcomes, and inflammatory marker resolution.

## Materials and methods

Ethics

This study was approved by the Institutional Ethics Committee (IEC) of Apollo Hospitals, Bengaluru (IEC Bio‑Medical Research Application no: AHB-BMR-018/08-212). The need for informed consent was waived because of the retrospective design of the study. This work conformed to the requirements of the Declaration of Helsinki, 1964, and its subsequent amendments.

Study design

This was a retrospective observational study conducted at Apollo Hospitals, Bengaluru, India, a tertiary care hospital. The patients' data between March 1, 2021, and June 1, 2021, were collected from the hospital information system.

Inclusion and exclusion criteria

Adult patients (aged >18 years) of both genders with laboratory-confirmed COVID-19 via reverse transcription-polymerase chain reaction from nasopharyngeal swab or sputum with raised inflammatory markers in serum (interleukin-6, IL-6, level of >40 pg/mL and two of the following criteria: C-reactive protein, CRP, level of >100 mg/L, D-dimer level of >1,000 ng/mL, ferritin level of >500 ng/mL, and lactate dehydrogenase, LDH, level of >300 U/L) who were likely to progress into cytokine storm or hyperinflammatory response, as well as critically ill patients (critically ill was defined by quick sequential organ failure assessment, qSOFA, >2, CURB-65 >2, or oxygen requirement >15 L/minute) even without raised inflammatory markers in serum, were included [14,15]. Patients with raised serum procalcitonin or troponin levels indicating cardiac injury or concurrent infections; those with a history of gastrointestinal (GI) bleeding, active malignancies, and active fungal, bacterial, or viral (hepatitis B virus or human immunodeficiency virus) infections; those who received immunosuppressive drugs; and pregnant and lactating women were excluded.

Treatment

The patients were divided into two groups matched by age, gender, and disease (COVID-19) severity qSOFA and CURB-65 score] as follows: 1) Group A: pulse methylprednisolone therapy (Table 1) plus standard treatment for COVID-19, and 2) Group B: standard treatment for COVID-19.

**Table 1:** Dose and duration of methylprednisolone pulse therapy in Group A The results are represented by the number (percentage)

Dose	Survivors (n = 79)	Nonsurvivors (n = 11)
125 mg twice daily for 3 days	2 (2.5)	0 (0)
250 mg twice daily for 3 days	65 (82.3)	10 (90.9)
>250 mg twice daily for 3 days	12 (15.2)	1 (9.1)

In Group A, dose selection (125, 250, and >250 mg) was guided by severity indicators and IL-6 thresholds, per institutional protocol. Pulse steroids were discontinued after three days without tapering. The standard treatment for COVID-19 included antipyretic (paracetamol), antimicrobials (azithromycin, doxycycline, and ivermectin), antivirals (remdesivir), anticoagulants (low-molecular-weight heparin), anti-inflammatory agents (tocilizumab), and convalescent plasma as per institutional protocol. Standard treatment varied based on individual clinical status. Medications used for other comorbidities were recorded.

Variables and outcomes

The demographic characteristics (age, gender, smoking status, comorbidities, and Charlson comorbidity index) of the two groups of patients were collected at baseline (at admission only). Controls were manually matched 1:1 based on age, gender, and disease severity (qSOFA and CURB-65), without the use of propensity score matching. The clinical status was ascertained in terms of symptoms, body temperature, respiratory rate, blood pressure, qSOFA, CURB-65 score, and time from illness onset to hospitalization. The following laboratory parameters were evaluated: complete hemogram, CRP level, D-dimer level, IL-6 level, ferritin level, and LDH level. Pulmonary involvement was evaluated by chest X-ray, Borg score, SpO_2_, and the need and mode of oxygen support.

The primary outcomes were the mortality rate at Day 3 and the duration of hospitalization in the two groups. Day 3 mortality was chosen to assess the immediate clinical effect of pulse methylprednisolone therapy, which was administered over a three-day course. This timing aligns with the end of the treatment window, allowing for evaluation of the therapy's early impact on survival in patients at risk of cytokine storm. The secondary outcomes were the number of patients who were discharged at the end of treatment, need for oxygen support, duration of intensive care unit (ICU) stay, SpO_2_, oxygen requirement, body temperature, respiratory rate, blood pressure, and laboratory parameters (complete hemogram, CRP level, D-dimer level, IL-6 level, ferritin level, and LDH level) in the two groups.

These secondary outcomes were partially prespecified, focusing on clinically relevant markers of inflammation and respiratory function; however, a few variables, such as blood pressure and respiratory rate, were included in the analysis in an exploratory capacity based on data availability and relevance to disease severity. All adverse reactions were also recorded.

Statistical analyses

The normality of the data was checked by the Kolmogorov-Smirnov test. The results are represented by the mean ± standard deviation, median (range), or number (percentage) as applicable. The categorical variables were compared by chi-square test, while the continuous variables were compared by unpaired t-test or Mann-Whitney test, as applicable. No multivariable regression analyses were performed; all comparisons were unadjusted. The two groups were matched on key baseline variables (age, gender, and disease severity) to minimize confounding; however, we acknowledge that residual confounding cannot be excluded due to the observational design. All analyses were conducted using Statistical Package for the Social Sciences version 23 (IBM Corp., Armonk, NY). A p value of <0.05 was considered statistically significant.

## Results

During the study period of three months, a total of 90 patients with severe COVID-19 were treated with pulse methylprednisolone therapy (Group A). The data of the corresponding 90 patients matched by age, gender, and disease severity were retrieved (Group B). The demographic, clinical, and laboratory parameters of both groups were comparable at baseline on the day of admission (Table 2).

**Table 2:** Demographic and clinical characteristics of the patients on the day of admission The results are represented by the mean ± standard deviation, median (range), or number (percentage) ^*^p < 0.05 vs. corresponding Group A values; ^#^Unpaired t-test; ^@^Chi-square test; ^$^Mann-Whitney U test BIPAP: bilevel positive airway pressure; CCI: Charlson comorbidity index; NRBM: non-rebreather mask; qSOFA: quick sequential organ failure assessment; TLC: total leucocyte count

Parameters	Group A (n = 90)	Group B (n = 90)	p value
Age (years)	62.7 ± 8.5	63.5±7.2	0.006^*,#^
Gender (male)	61 (67.8)	59 (65.6)	0.874^@^
Smoker	20 (22.2)	10 (11.1)	0.072^@^
Comorbidities			
None	21 (23.3)	26 (28.9)	0.438^@^
Diabetes	37 (41.1)	39 (43.3)	0.877^@^
Hypertension	26 (28.9)	24 (26.7)	0.859^@^
Obesity	31 (34.4)	30 (33.3)	0.872^@^
Cardiovascular diseases	4 (4.4)	2 (2.2)	0.681^@^
Chronic liver diseases	2 (2.2)	3 (3.3)	1.000^@^
Chronic renal diseases	3 (3.3)	6 (6.6)	0.496^@^
Chronic lung diseases	4 (4.4)	3 (3.3)	1.000^@^
Malignancies in remission	1 (1.1)	1 (1.1)	1.000^@^
CCI index	2 (0-4)	2 (1-4)	0.312^$^
Symptoms			
Fever	75 (83.3)	77 (85.6)	0.827^@^
Dyspnea	48 (53.3)	45 (50.0)	0.738^@^
Cough	62 (68.9)	62 (68.9)	1.000^@^
Myalgia	12 (13.3)	7 (7.8)	0.323^@^
Headache	6 (6.6)	5 (5.5)	1.000^@^
Gastrointestinal symptoms	5 (5.5)	6 (6.6)	1.000^@^
Generalized weakness	38 (42.2)	36 (40.0)	0.881^@^
Body temperature (°C)	99.3 (97-101.1)	100.1 (98.0-104.1)	0.091^$^
Respiratory rate (per minute)	20 (18-24)	21 (18-22)	0.341^$^
Respiratory rate of >22/minute	33 (36.7)	28 (31.1)	0.540^@^
Heart rate (per minute)	84.6 ± 15.3	84.7 ± 13.8	0.675^#^
Heart rate of >100/minute	21 (23.3)	24 (26.7)	0.712^@^
Systolic blood pressure (mm Hg)	128.1 ± 18.5	125.9 ± 16.4	0.029^*,#^
Systolic blood pressure of <100 mmHg	2 (2.2)	4 (4.4)	0.681^@^
Diastolic blood pressure (mmHg)	76.1 ± 9.4	74.0 ± 14.2	0.058^#^
qSOFA score	0.9 (0.7-1.4)	1.0 (0.7-2.4)	0.212^$^
CURB-65 score	1.6 (0.9-2.3)	1.5 (1.1-2.5)	0.456^$^
Time from illness onset to hospitalization (days)	6.4 ± 2.3	6.3 ± 1.9	0.758^#^
Laboratory parameters			
TLC (×10^3^/µL)^$^	7.6 (3.0-18.4)	6.4 (3.3-20.8)	0.758^$^
TLC of 4-10 × 10^3^/µL	23 (25.6)	32 (35.6)	0.736^@^
TLC of <4 × 10^3^/µL	10 (11.1)	6 (6.6)	0.042^*,@^
TLC of >10 × 10^3^/µL	67 (74.4)	54 (60.0)	0.023^*,@^
Lymphocyte count (%)	23 (6-44)	26 (4-48)	0.213^$^
Lymphocyte count of 20-40%	12 (13.3)	11 (12.2)	0.999^@^
Lymphocyte count of <20%	74 (82.2)	62 (68.9)	0.063^@^
Lymphocyte count of >40%	6 (6.6)	10 (11.1)	0.307^@^
Platelet count (×10^3^/µL)	167 (153-235)	174 (168-258)	0.288^$^
Platelet count of <150 × 10^3^/µL	8 (8.8)	6 (6.6)	0.774^@^
Platelet count of 150-450 × 10^3^/µL	73 (81.1)	82 (91.1)	0.089^@^
Platelet count of >450 × 10^3^/µL	10 (11.1)	2 (2.2)	0.036^*,@^
Hemoglobin level (g/dL)	12.1 (8.3-15.7)	10.1 (9.4-12.4)	0.022^*,$^
C-reactive protein level (mg/L)	150.4 (67.6-200.2)	154.1 (60.9-265.2)	0.882^$^
D-dimer level (ng/mL)	10.4 (0.08-18.79)	9.6 (0.27-10.0)	0.615^$^
Interleukin-6 level (pg/mL)	54.8 (11.12-120.8)	64.8 (17.6-123.15)	0.224^$^
Ferritin level (ng/mL)	564.7 (37.3-1,532.0)	579.6 (359.5-1,466.0)	0.476^$^
Lactate dehydrogenase level (U/L)	578 (176-1,255)	599 (168-830)	0.736^$^
Pulmonary involvement			
Borg score	7.7 ± 2.3	7.1 ± 3.1	0.736^#^
Oxygen saturation, SpO_2_ (%)	89 (80-97)	83 (79-94)	0.042^*,$^
Need for oxygen support	81 (90.0)	89 (98.9)	0.023^*,@^
Mode of oxygen delivery			
Nasal cannula	5 (5.5)	9 (10.0)	0.404^@^
Simple mask	64 (71.2)	13 (14.4)	0.001^*,@^
NRBM	4 (4.4)	18 (20.0)	0.003^*,@^
Noninvasive ventilation (BIPAP)	17 (18.9)	50 (55.6)	0.001^*,@^
Oxygen requirement (L/minute)	6.5 (2-15)	8.8 (6-15)	0.004^*,$^
Ground-glass opacity in chest X-ray			
Unilateral	52 (57.8)	39 (43.3)	0.059^@^
Bilateral	38 (42.2)	51 (56.7)	-
Lung consolidation in chest X-ray			
Unilateral	59 (65.6)	43 (47.8)	0.021^*,@^
Bilateral	31 (34.4)	47 (52.2)	-
Pulmonary involvement in chest X-ray			
Mild (<8%)	16 (17.8)	17 (18.9)	-
Moderate (8-15%)	22 (24.4)	20 (22.2)	0.932^@^
Severe (15-25%)	52 (57.8)	53 (58.9)	-

The frequencies of comorbidities, presenting symptoms, and disease severity status (qSOFA, CURB-65 score, pulmonary involvement, and time from illness onset to hospitalization) were similar between the two groups. There were, however, significant differences in the smoking status and the distribution of the mode of oxygen delivery between the two groups. Smoking status and hemoglobin levels were not included in the original matching criteria, which may have introduced residual confounding; this limitation has been acknowledged in the study.

**Figure 1:**
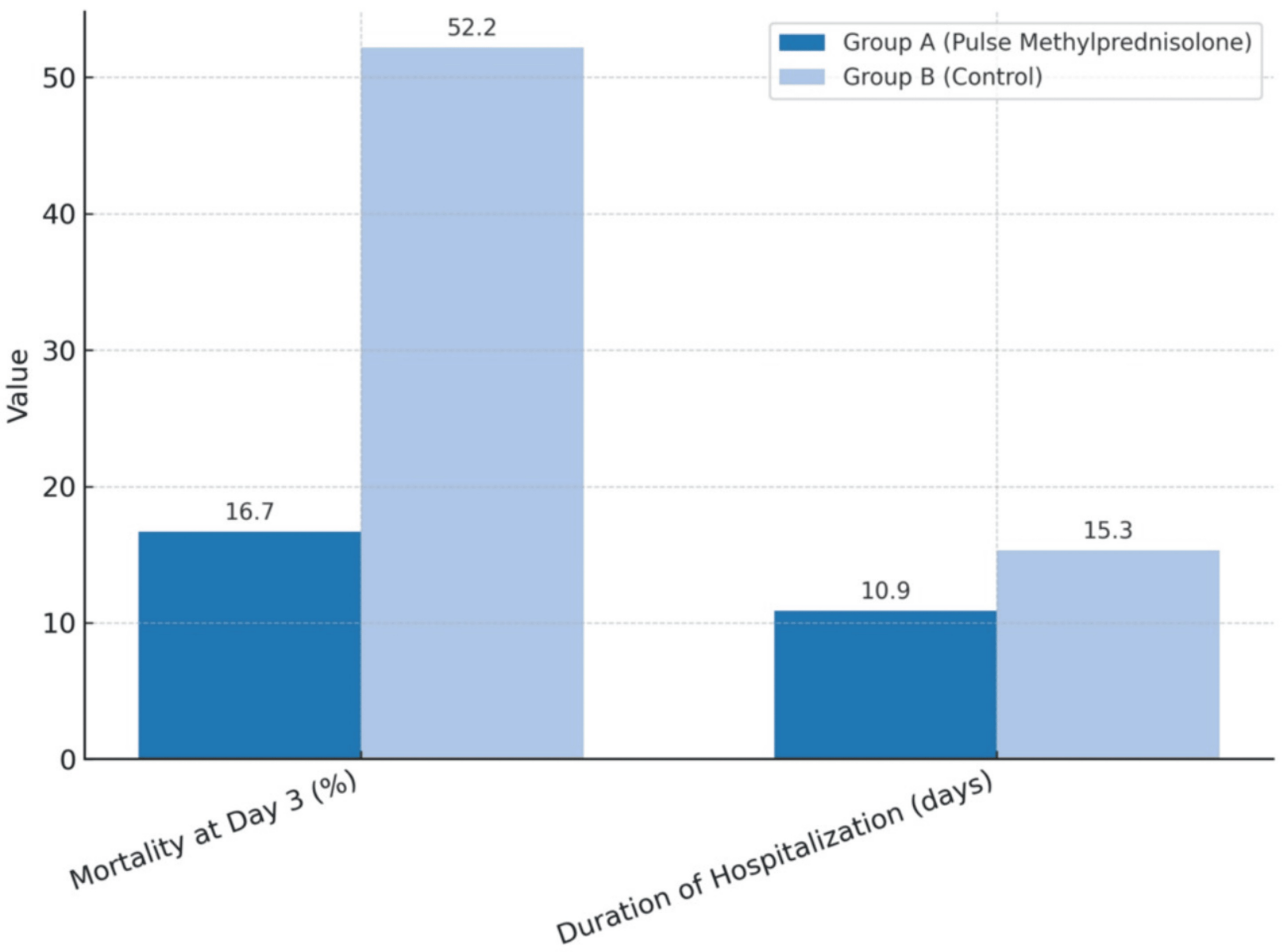
Comparison of mortality at Day 3 and duration of hospitalization between Groups A and B

At Day 3, there were significant differences in the mortality rate (16.7% vs. 52.2%) between Groups A and B. Also, there was a significant difference in the duration of hospitalization, 10.9 (3-22) vs. 15.3 (1-30) days, between Groups A and B (Figure 1).

Pulse methylprednisolone therapy significantly improved the recovery (discharge) rate, need for high-volume oxygen support, and the duration of ICU stay compared to standard treatment alone (Table 3).

**Table 3:** Outcomes of the two groups of patients The results are represented by the median (range) or number (percentage) ^*^p < 0.05 vs. corresponding Group A values; ^$^Mann-Whiney U test; ^@^Chi-square test ICU: intensive care unit

Outcomes	Group A (n = 90)	Group B (n = 90)	p value
Number of patients who died at day 3	15 (16.7)	47 (52.2)	0.001^*,@^
Duration of hospitalization (days)	10.9 (3-22)	15.3 (1-30)	0.001^*,$^
Number of patients who were discharged at the end of treatment	75 (83.3)	43 (47.8)	0.001^*,@^
Need for oxygen support	86 (95.6)	89 (98.9)	0.277^@^
Need for high-volume oxygen support	49 (54.4)	57 (63.3)	0.243^@^
ICU admission	27 (30.0)	28 (31.1)	0.868^@^
Duration of ICU stay (days)	5.7 (1-19)	7.5 (1-22)^*^	0.004^*,$^

A total of 27 patients (30%) in Group A and 28 patients (31.1%) in Group B were admitted to the ICU during their hospitalization. The analysis of ICU duration was conducted exclusively among patients who required ICU care. In this subgroup, the median duration of ICU stay was significantly shorter in Group A compared to Group B: 5.7 days (range 1-19) vs. 7.5 days (range 1-22). The comparison of the clinical and laboratory parameters of the patients at the end of treatment and discharge/death is enumerated in Table 4. There were significant improvements in SpO_2_ and oxygen requirement between the two groups at the end of treatment and discharge/death. Group A had significantly lower bilevel positive airway pressure (BiPAP) use (20% vs. 3.3%) but similar mechanical ventilation rates. This suggests a reduction in progression to noninvasive ventilation among patients receiving pulse methylprednisolone. Although mechanical ventilation rates were similar, the lower need for BiPAP indicates earlier resolution of hypoxia. Pulse methylprednisolone therapy significantly improved CRP level, D-dimer level, IL-6 level, ferritin level, and LDH level compared to the standard treatment alone. There were significant improvements in these parameters at the end of treatment and discharge/death compared to the corresponding previous values (Table 4). The changes were more prominent in Group A than in Group B. Heart rate and respiratory rate fluctuations were likely influenced by multiple factors, including oxygen weaning, underlying lung injury, and ongoing inflammatory process.

**Table 4:** Clinical and laboratory parameters of the patients at the end of treatment and discharge/death The results are represented by the mean ± standard deviation, median (range), or number (percentage) ^*^p < 0.05 vs. corresponding Group A values; ^†^p < 0.05 vs. corresponding values at the end of treatment; ^‡^p < 0.05 vs. corresponding values at baseline (on the day of admission); ^$^Mann-Whitney U test; ^@^Chi-square test; ^#^Unpaired t-test BIPAP: bilevel positive airway pressure; NRBM: non-rebreather mask

Parameter	At the end of treatment		p value	At discharge/death		p value
	Group A (n = 90)	Group B (n = 90)		Group A (n = 90)	Group B (n = 90)	
Oxygen saturation, SpO_2_ (%)^$^	92 (82-96)	86 (74-88)^*^	0.001^*^	96 (92-98)^‡^	92 (88-96)^*,†,‡^	0.001^*^
Oxygen requirement (L/minute)^$^	10 (4-15)^‡^	12 (6-15)^‡^	0.015^*^	4.8 (4-10)^†^	8 (4-12)^*^	0.002^*^
Mode of oxygen delivery						
Nasal cannula^@^	2 (2.2)^‡^	0 (0)^*,‡^	0.04^*^	10 (11.1)^†,‡^	4 (4.4)^*,†,‡^	0.03^*^
Simple mask^@^	39 (43.3)^‡^	28 (31.1)^*,‡^	-	36 (40.0)^‡^	30 (33.3)^‡^	-
NRBM mask^@^	42 (46.7)^‡^	48 (53.3)^‡^	-	28 (31.1)^†,‡^	40 (44.4)^*,†,‡^	-
High-flow nasal cannula^@^	8 (88.9)	10 (11.1)	-	3 (3.3)^†^	4 (4.4)^†^	-
Noninvasive ventilation (BIPAP)^@^	18 (20.0)	32 (35.6)^*,‡^	-	3 (3.3)^†,‡^	10 (11.1)^*,†,‡^	-
Mechanical ventilation (invasive)^@^	14 (15.6)	20 (22.2)^*^	-	16 (17.8)	20 (22.2)^*^	-
Systolic blood pressure (mmHg)^#^	127.6 ± 15.3	122.8 ± 20.0^‡^	0.048^*^	122.8 ± 20.0	126 ± 14.0^‡^	0.26
Diastolic blood pressure (mmHg)^#^	78.0 ± 8.8	74.0 ± 14.2	0.09	74.0 ± 14.2	74.7 ± 9.0	0.72
Heart rate (per minute)^#^	82.0 ± 11.9	82.0 ± 11.9	1.000	84.7 ± 13.8	81.2 ± 7.9	0.08
Respiratory rate (per minute)^#^	20.8 ± 3.1	18.6 ± 2.4^‡^	0.001^*^	23.1 ± 5.1	21.6 ± 2.4^‡^	0.03^*^
Body temperature (°C)^#^	97.8 + 2.0	98.2 + 3.2	0.40	97.8 + 1.4	97.6 + 2.4	0.68
Laboratory parameters						
Total leucocyte count (×10^3^/µL)^$^	10.6 (6.8-19.5)^‡^	12.8 (6.9-20.2)^‡^	0.07	9.5 (6.2-16.8)^‡^	10.6 (5.9-18.8)^‡^	0.14
Lymphocyte count (%)^$^	26 (3-34)	25 (4-23)	0.95	24 (3-20)	26 (3-13)	0.79
Platelet count (×10^3^/µL)^$^	271 (160-441)^‡^	243 (223-423)^‡^	0.11	252 (204-406)^‡^	193 (252-380)^*,†,‡^	0.01^*^
Hemoglobin level (g/dL)^$^	11.4 (10-14.6)	13.1 (9.6-16.2)	0.03^*^	12.6 (10.2-14.6)	10.7 (8.6-14.6)	0.04^*^
C-reactive protein level (mg/L)^$^	40.8 (13.1-209.5)^‡^	91.9 (31.8-575.9)^*,‡^	0.001^*^	30.6 (19.0-139.7)^†,‡^	77.4 (23.2-86.7)^*,†,‡^	0.003^*^
D-dimer level (ng/mL)^$^	1.2 (0.4-8.8)^‡^	1.9 (0.2-5.2)^*,‡^	0.02^*^	0.3 (0.2-6.2)^†,‡^	0.5 (0.2-5.2)^*,†,‡^	0.04^*^
Interleukin-6 level (pg/mL)^$^	32.5 (1.5-154.9)^‡^	39.3 (6.8-170.4)^*,‡^	0.08	-	-	-
Ferritin level (ng/mL)^$^	480.9 (78.1-1,178.3)^‡^	559.2 (97.4-852.3)^*,‡^	0.04^*^	-	-	-
Lactate dehydrogenase level (U/L)^$^	351.6 (152.6-772.6)^‡^	438.2 (201.6-772.8)^*,‡^	0.01^*^	-	-	-

The distribution of the use of other drugs (standard treatment) was similar in both groups. There were no changes in the use of medications for other comorbidities, including insulin and oral hypoglycemic agents in both groups, implying that pulse methylprednisolone therapy did not adversely impact the glycemic status of the patients; however, due to the lack of systematic blood glucose monitoring, no definitive conclusion regarding glycemic safety can be made. Pulse methylprednisolone therapy was safe, and no immediate adverse events (e.g., arrhythmias, uncontrolled hypertension, and GI bleeding) were reported; however, formal monitoring protocols were limited due to the retrospective design. Long-term adverse effects such as secondary infections or hyperglycemia were not assessed.

## Discussion

In this study, we found that adjunct pulse methylprednisolone therapy could significantly improve the mortality rate, duration of hospitalization, recovery rate, SpO_2_, oxygen requirement, and laboratory parameters (CRP, D-dimer, IL-6, ferritin, and LDH levels) compared to standard treatment alone in patients with severe COVID-19 at risk of hyperinflammatory response. This illustrates the overall beneficial effects of pulse methylprednisolone therapy in these patients.

Pulse therapy with methylprednisolone is frequently used in conditions where it is important to exert a quick immunosuppressive effect [16]. Corticosteroids inhibit the migration of leukocytes to inflamed tissues, thereby facilitating their migration from the bone marrow to the blood and inhibiting apoptosis of leukocytes. They also inhibit leukocyte reactive oxygen species, increase IL-10, and alter the maturation and differentiation of dendritic cells. Corticosteroids also modulate natural killer cytolytic activity and monocyte activation, as well as downregulate IL-1, IL-2, IL-6, IL-8, interferon-γ, and tumor necrosis factor-α by transrepression [17,18].

Low concentrations of corticosteroids (≤7.5 mg/day) mediate effects via genomic events regulating the transcription of proinflammatory molecules. Medium concentrations (7.5-30 mg/day) activate both genomic and nongenomic events. Very high concentrations (>100 mg/day) intercalate into cellular membranes, jeopardize cation transport through the plasma membrane, and leak protons from the mitochondria. As the dose of corticosteroid increases and the receptors are saturated, nongenomic effects are more prominent. With pulse dosing (500-1,000 mg/day), corticosteroid-induced apoptosis is noted. This explains the very rapid immunosuppressive and anti-inflammatory effects of pulse corticosteroid therapy. In the early stage of inflammatory cytokine storm in COVID-19, pulse methylprednisolone therapy could alter the disease outcome, as seen in our patients. This is due to the nongenomic event, wherein the anti-inflammatory activity of methylprednisolone is maximum, while the risk of adverse effects is low [19,20].

Previous reports have demonstrated the beneficial effects of pulse methylprednisolone therapy in patients with severe COVID-19 [21,22]. It was found that COVID-19 patients up to 70 years of age have the best response to pulse methylprednisolone therapy [23]. A single-blind, randomized controlled clinical trial involving severe hospitalized COVID-19 patients showed that pulse methylprednisolone could be used for the treatment [11]. In another observational study involving 318 COVID-19 patients, pulse methylprednisolone therapy was found to be effective in increasing the survival rates of patients at risk of developing hyperinflammatory response [15]. In another study involving 216 patients, high-dose methylprednisolone for three days followed by oral prednisone for 14 days, compared to 6 mg dexamethasone for 7-10 days, significantly reduced the recovery time, the need for transfer to ICU, and the severity markers in the serum (CRP, D-dimer, and LDH levels) [24]. In another study, a short course of pulse methylprednisolone therapy was found to be effective in improving the prognosis of patients with COVID-19 pneumonia with features of hyperinflammatory response and respiratory deterioration [25]. Repeated pulses of methylprednisolone in adults hospitalized with COVID-19 pneumonia and ARDS were found to be beneficial [26]. A two-month mortality benefit of pulse methylprednisolone therapy was also established in severe COVID-19 [27].

The reduction in the serum level of inflammatory markers (CRP, D-dimer, IL-6, ferritin, and LDH levels) in our study, suggesting amelioration of hyperinflammatory response, following pulse methylprednisolone therapy, is consistent with findings of other studies. Elevated serum levels of IL-6 and CRP as inflammatory markers are associated with the severity of COVID-19 and can be used as a prognostic marker of the disease [28]. Specific IL-1 and IL-6 inhibitors (anakinra or tocilizumab) can be used in the treatment of severe COVID-19 complicated with respiratory failure. However, in certain COVID-19 patients, it is not possible to achieve a sustained improvement with the use of these drugs [29]. In these patients, pulse methylprednisolone therapy could be beneficial. Our patients were also treated with another anti-inflammatory agent (tocilizumab) and antithrombotic medication (low-molecular-weight heparin) to reduce the chance of cytokine storm and avoid disseminated intravascular coagulation [30,31]. However, the frequency of use of such drugs was comparable between the two groups, thereby further reinforcing the beneficial effects of adjunct pulse methylprednisolone therapy. A reduction in the oxygen requirement, improvement in SpO_2_, reduction in the duration of hospitalization and ICU stay, improvement in the recovery rate, and overall improvement in the mortality following pulse methylprednisolone therapy imply clinical improvement in these patients.

It is pertinent to mention that some contradictory results with pulse methylprednisolone therapy have also been reported in the literature. In a double-blinded, randomized, placebo-controlled trial, it was found that methylprednisolone (0.5 mg/kg twice daily for five days) did not reduce the 28-day mortality rates in COVID-19 patients on oxygen support. However, more critically ill patients were included in this study [32]. In another retrospective study, the mortality rates of severe COVID-19 patients were not different following administration of methylprednisolone 1 mg/kg daily and administration of methylprednisolone as pulse therapy [33].

There are limitations to this study. First, the duration of follow-up was small. Second, selection bias could not be completely ruled out for the selection of the controls, as no randomization was followed. Third, the sample size was small. Last, the retrospective design of the study carried its limitations. Data for IL-6, ferritin, and LDH at discharge were not consistently available due to limited resources and prioritization of clinical indicators during recovery. Notwithstanding these limitations, based on our findings, pulse methylprednisolone could be used for the treatment of severe COVID-19 patients at risk of hyperinflammatory response. Our results need to be confirmed by large-scale randomized controlled trials with a longer follow-up in the future.

## Conclusions

Adjunct pulse methylprednisolone therapy significantly improved mortality rate, duration of hospitalization, enhanced recovery rates, lowered oxygen requirements, and improved key inflammatory and hematological markers compared to standard treatment alone in patients with severe COVID-19 at risk of hyperinflammatory response. While the results suggest a potential clinical benefit of high- dose corticosteroid pulse therapy, the study is limited by its retrospective design and the absence of comprehensive adverse event monitoring. Future research should aim to validate these findings through large-scale randomized controlled trials and explore comparative effectiveness against other immunomodulatory agents, such as IL-6 inhibitors or standard- dose dexamethasone.
